# Supplementary material for: Cost-utility analysis of endoscopic lumbar discectomy following a uniform clinical pathway in the Korean national health insurance system
Source: PLoS One. 2023 Jun 15;18(6):e0287092. doi: 10.1371/journal.pone.0287092 (PMC10270587; doi:10.1371/journal.pone.0287092)
Supplement: Attachment — Submitted filename: Authors revision letter20220513.docx [file pone.0287092.s002.docx]

Author’s revision letter

Reviewer #1: This paper suffers from a lack of sample size. There appears to be some attempt of a design in the data analysis section on pages 11 and 12. However, these considerations are not clearly written and the justification for 28 subjects is not evident.

Answer.

I sincerely appreciate your critical comment. This study was a subgroup analysis of prospectively collected data, and the purpose of this study was to analyze the cost-utility of full-endoscopic lumbar discectomy. The number of patients was not pre-determined, and all patients with 2-year follow-up data were included. Although the number of patients was small, all patients underwent the same clinical pathway, surgical technique, and hospital costs incurred during admission. The operations were performed at a university hospital, and no additional uninsured surgical materials, such as a hemostatic agent or anti-adhesive agent, were used, except for the electrode for full endoscopic lumbar discectomy (FELD). This homogeneity of the data may compensate for the weakness of the small number of patients. Nonetheless, the small number of patients was the critical weakness of this study, and this limitation was clarified in the revised manuscript.

Page 17, line 6

This study has several limitations. First, it presents results from a small number of patients, which were not sufficient to fully represent the cost-utility of FELD. In addition, this study analyzed only direct medical costs, although indirect medical costs may increase medical expenditures as a whole. However, the main purpose of this study was to show the relevance of the costs incurred at hospitals for FELD. In this regard, we used homogeneous data from patients who underwent the same surgical procedure and were managed with the same clinical pathway in a single tertiary referral hospital. Although the number of patients was small, the surgical technique, medical costs, and postoperative management were homogeneous. In addition, the surgery was performed at a university hospital, and no additional uninsured surgical materials such as a hemostatic agent or anti-adhesive agent were used, except for the electrode for FELD. We acknowledge that the small number of patients was the major weakness of this study, but the homogeneity may compensate for this limitation.

The pre post analysis with the generalized mixed model approach is reasonable. However, again, the sample size does not appear adequate and there should have been some consideration of a comparison to the standard open microdiscectomy procedure for cost comparisons. The study is, at best, descriptive. A propensity matching procedure with adequate statistical power should have been attempted to truly determine the utility of the FELD procedure.

Answer.

I totally agree with your opinion. A comparative study with a sufficient number of patients should have been done to effectively show the cost-effectiveness of FELD. However, the open discectomies were performed at several other hospitals and the direct medical costs were variable among hospitals. The purpose of this study was to show the cost-utility of FELD following a uniform clinical pathway in the Korean national health insurance system and to present the actual costs of FELD to support a realistic reimbursement. In this regard, we included homogeneous data from a university hospital that strictly follows the regulations of the national health insurance system and minimizes the use of un-insured surgical materials. Nonetheless, a comparative study should have been attempted to truly determine the utility of the FELD procedure. We have acknowledged this limitation and described it in the limitations section.

Page 17, line 21

Last, but not least, FELD was not compared to standard open discectomy, which would have made it possible to truly determine the utility of FELD. However, open discectomies were performed at several hospitals, and the direct medical costs such as hospital stay, room charges, magnetic resonance imaging, and uninsured surgical materials were variable. Therefore, the need to preserve homogeneity meant that open discectomy could not be compared with FELD in this study.

Reviewer #2: Nice paper in a gray error of the literature
- Sample size is a bit small

Answer.

I sincerely appreciate your critical comment. This study was a subgroup analysis of prospectively collected data, and the purpose of this study was to analyze the cost-utility of full-endoscopic lumbar discectomy. The number of patients was not pre-determined, and all patients with 2-year follow-up data were included. Although the number of patients was small, all patients underwent the same clinical pathway, surgical technique, and hospital costs incurred during admission. The operations were performed at a university hospital, and no additional uninsured surgical materials, such as a hemostatic agent or anti-adhesive agent, were used, except for the electrode for full endoscopic lumbar discectomy (FELD). This homogeneity of the data may compensate for the weakness of the small number of patients. Nonetheless, the small number of patients was the critical weakness of this study, and this limitation was clarified in the revised manuscript.

Page 17, line 6

First, it presents results from a small number of patients, which were not sufficient to fully represent the cost-utility of FELD. In addition, this study analyzed only direct medical costs, although indirect medical costs may increase medical expenditures as a whole. However, the main purpose of this study was to show the relevance of the costs incurred at hospitals for FELD. In this regard, we used homogeneous data from patients who underwent the same surgical procedure and were managed with the same clinical pathway in a single tertiary referral hospital. Although the number of patients was small, the surgical technique, medical costs, and postoperative management were homogeneous. In addition, the surgery was performed at a university hospital, and no additional uninsured surgical materials such as a hemostatic agent or anti-adhesive agent were used, except for the electrode for FELD. We acknowledge that the small number of patients was the major weakness of this study, but the homogeneity may compensate for this limitation.
Introduction is well written
The outcomes of open discectomy and endoscopic discectomy are similar, but physicians should inform to patients about all possible options and respect their choices--> cite Gadjradj PS, Rubinstein SM, Peul WC, Depauw PR, Vleggeert-Lankamp CL, Seiger A, van Susante JL, de Boer MR, van Tulder MW, Harhangi BS. Full endoscopic versus open discectomy for sciatica: randomised controlled non-inferiority trial. BMJ. 2022 Feb 21;376:e065846. doi: 10.1136/bmj-2021-065846. PMID: 35190388.

Answer.

Thank you very much for the recommendation. We included and discussed that valuable article in the present study.

Economic acceptability is usually judged by willingness to pay (WTP), but WTP for FELD has not yet been determined--> it has now from the Netherlands

Gadjradj PS, Broulikova HM, van Dongen JM, Rubinstein SM, Depauw PR, Vleggeert C, Seiger A, Peul WC, van Susante JL, van Tulder MW, Harhangi BS. Cost-effectiveness of full endoscopic versus open discectomy for sciatica. Br J Sports Med. 2022 Feb 20:bjsports-2021-104808. doi: 10.1136/bjsports-2021-104808. Epub ahead of print. PMID: 35185010.

Answer.

I appreciate your comment and recommendation of those articles. As the recommended study showed, a comparative cost-effective analysis will be necessary to include endoscopic lumbar discectomy in the package of health insurance. Although this study was not designed like the recommended article, the recommended articles were included as important references in this study.

Page 5, line 19

A recent randomized controlled multi-center trial by Gadjradj et al. showed better clinical outcomes after FELD than after open discectomy, and the cost of surgery was lower with FELD than with open discectomy.[4,16] Economic acceptability is usually judged by willingness to pay (WTP), and those studies showed the WTP for FELD.[4,16] The results enabled FELD to be included in the health insurance package of the Netherlands.

The choice among surgical options would be fairer if the actual costs of those supplies were reimbursed, but WTP for FELD has not yet been determined in the ROK.[8-10]

Page 16, line 14

Recently, Gadjradj et al. showed better leg pain and QALYs after endoscopic lumbar discectomy than after open discectomy.[4,16] through a randomized controlled trial. This study showed that the WTP for endoscopic lumbar discectomy will be similar or higher than the WTP of 6112 Euros ($6612) in the Netherlands.[4,16] Those results prompted the Dutch Ministry of Health to include endoscopic lumbar discectomy in the basic health insurance package.[4,16]
Please explain perc adhesiolysis: did all consertvative care patients receieve it? Did all surgery patients receive it prior?

Answer.

I appreciate the comment. As you pointed out, adhesiolysis is not a routine procedure and choice of non-surgical treatment was at the discretion of doctors. The sentence may confuse readers, so it was deleted.

Rephrase clinical pathyway

Answer.

Thank you for giving us a chance to introduce the clinical pathway. It was rephrased as follows.

Page 8, line 23

**Clinical pathway of endoscopic lumbar discectomy**

All patients were managed with the same protocol and orders (clinical pathway), which were developed by the clinicians.[28] When the decision was made to perform surgery, basic preoperative check-ups such as blood tests, electrocardiography, chest X-rays, and spinal X-rays were performed at the outpatient clinic. On day 1 (preoperative day 1), the patient was admitted in the afternoon for a preoperative check-up by an anesthesiologist and other physicians, and provided informed consent for surgery. On day 2 (the day of surgery), the patient was transferred to the operation room after a sensitivity test for antibiotics (first-generation cephalosporin) and underwent surgery under general anesthesia. A Foley catheter was not inserted. After the operation, the patient was moved to the post-anesthesia care unit and was returned to the general ward after fully awakening, as confirmed by an anesthesiologist. The patient was encouraged to stand up and walk around the hall of the general ward with a walking aid within 1 hour of returning to the general ward. Pain was controlled with oral acetaminophen and a regular diet was allowed in the evening after confirmation of bowel sound or passing flatussounds. Postoperative magnetic resonance imaging (MRI) was taken during the evening or night to check the results of discectomy. On day 3 (postoperative day 1), the patient was discharged home in the afternoon after confirmation of independent ambulation, improvements of preoperative symptoms/signs and the absence of any postoperative complication such as a neurological problem. After discharge, a lumbar-supporting brace was not applied, and office work was encouraged within 1 month after surgery. However, strenuous activities such as sports, vigorous leisure activities, and weightlifting were not allowed until 3 months after surgery. The patient was scheduled to visit the outpatient clinic at postoperative 1, 3, 6, and 12 months and yearly thereafter. If pain recurred, it was managed with drugs and/or epidural injections on an outpatient basis. If the pain was unbearable or those measures were not effective for 2 months, MRI was recommended to check for the possibility of recurrence.

Please explain the rationale for performing surgery under general anesthesia. I know some do this, but why do the authors do this?

Answer.

Thank you for this relevant comment. Many surgeons perform endoscopic surgery under local or regional anesthesia. I started to learn endoscopic lumbar discectomy from a surgeon who performed it under general anesthesia (Dr. Sebastian Ruetten in St. Anna Hospital, Germany). I also know that many surgeons prefer local or regional anesthesia to be able to receive feedback from patients. Moreover, postoperative recovery is better with local or regional anesthesia than general anesthesia, and hospital costs could be reduced by local or regional anesthesia. However, it was not easy to change my routine practice, and I use intraoperative neuromonitoring and the clinical pathway for those purposes. I clarified this point in the discussion section as follows.

Page 16, line 28

Although FELD was performed under general anesthesia in the current study, FEED could be effectively performed under local or regional anesthesia. Using local or regional anesthesia may enhance the safety and cost-effectiveness of FELD by reducing the costs of anesthesia, the time for recovery from anesthesia, and possibly the hospital stay.[45-47]

Was TELD not even attempted at L5-S1?

Answer.

There is evidence of equal efficacy of the transforaminal and interlaminar approach at L5-S1 and the selection of the approach was at the surgeon’s discretion. I prefer the interlaminar approach for L5-S1 level, and a transforaminal approach was not attempted.

Describe when the clinical parameters were measured.

Answer.

Thank you for the comment. The schedule was described in the revised manuscript.

Page 8, line 24

All participants were asked to complete patient-reported outcome questionnaires before the operation and at postoperative 1, 3, 6, 12, and 24 months

please explain what this electrode is used for.

Answer.

Thank you for the comment. We described the usage of the electrode in the introduction section.

Page 6, line 3

The electrode is an especially essential tool in FELD. It is specifically designed for the surgeon to navigate the tip of the electrode into the spinal canal through a spinal endoscope, and it is used for coagulation, shrinkage of tissue, and dissection.

What about the costs for reoperations?

Answer.

In the ROK, the cost of a reoperation is the same as the cost of the first operation. The cost of the operation is set according to the procedure of surgery, and the difficulty of revision surgery is not considered.

The ODI is usually presented as a range from 0 to 100

Answer.

I appreciate your comment and the score was presented as a range from 0 to 100 in the revised manuscript. Please see the changes throughout the manuscript.

Please create a table in which the costs are broken down: surgery, primary care, medication etc.

Answer.

Thank you for the suggestion. The costs were broken down into the costs of surgery, room, electrode, and general care. Among these costs, the costs of the room and magnetic resonance imaging were variable, and they were described as follows.

Table 3. Itemized costs at postoperative 1 month

| Itemized cost | Cost ($) |
| --- | --- |
| Surgery | 625 |
| Electrode | 700 |
| Anesthesia | 155 |
| Room (mean ± SD) | 95±97 |
| Single* | 3 |
| Twin* | 10 |
| Shared* | 15 |
| MRI (mean ± SD) | 867±311 |
| Pre-op* | 7 |
| Post-op* | 28 |
| Meal | 16 |
| Total cost, mean (95% CI)† | 3,255 (2,974, 3,536) |

*Number of patients.

SD, standard deviation; MRI, magnetic resonance imaging; Pre-op, preoperative; Post-op, postoperative.

†Total included the costs of itemized costs and other non-itemized costs such as general care and nursing.

Discuss the recently published trial by Gadjradj et al in Br J Sports Med.

Answer.

Thank you for recommending these relevant articles; they were discussed below as follows.

Page 5, line 19

A recent randomized controlled multi-center trial by Gadjradj et al. showed better clinical outcomes after FELD than after open discectomy, and the cost of surgery was lower with FELD than with open discectomy.[4,16] Economic acceptability is usually judged by willingness to pay (WTP), and those studies showed the WTP for FELD.[4,16] The results enabled FELD to be included in the health insurance package of the Netherlands.[4,16]

Page 6, line 6

The choice among surgical options would be fairer if the actual costs of those supplies were reimbursed, but WTP for FELD has not yet been determined in the ROK.[8-10]

Page 16, line 15

Recently, Gadjradj et al. showed better leg pain and QALYs after endoscopic lumbar discectomy than after open discectomy.[4,16] through a randomized controlled trial. This study showed that the WTP for endoscopic lumbar discectomy will be similar or higher than the WTP of 6112 Euros ($6612) in the Netherlands.[4,16] Those results prompted the Dutch Ministry of Health to include endoscopic lumbar discectomy in the basic health insurance package.[4,16]

Reviewer #3: Dear authors,
I congratulate your efforts for a detailed cost-utility analysis of the FELD procedure to be made available as a choice of surgery to the people ROK based on the QALY gained by the procedure objectively thereby improving the medical services provided to the affected population.

I have a few concerns that needs clarification before this manuscript could be considered for publication.

Rather than utilizing the cost of the electrode alone in the reimbursement calculation you can give an objective model where the hospital offering the tratement need not bear the brunt of the choice of the patient due to the financial constraints involved in the procedure like installation of FELD system, consumables etc., So that a practical statement can be made to the NHIS from this analysis for the recommended method and means of calculating the appropriate reimbursement so that the procedure is beneficial both to the service provider and the patient involved in it.

Answer.

I totally agree with your opinion, and hope to adopt the system in National Health Insurance system (NHIS). However, the goal of the Korean NHIS is to provide the same level of medical service to all patients irrespective of their wealth or insurance status. So far, not all hospital costs are covered by NHIS and un-insured costs are borne by patients. The government has been taking steps to gradually include all possible medical services under the control of NHIS, such as magnetic resonance imaging, and has attempted to control the inflation of medical costs by the NHIS regulations. Therefore, your model may not precisely suit the current NHIS of the ROK.

If needed, a comparison can be made between the open discectomy group and the FELD group to establish the superiority of the treatment method over the conventional gold standard procedure currently in use.

Answer.

I totally agree with your opinion. A comparative study with a sufficient number of patients should have been done to effectively show the cost-effectiveness of FELD. However, the open discectomies were performed at several other hospitals and the direct medical costs were variable among hospitals. The purpose of this study was to show the cost-utility of FELD following a uniform clinical pathway in the Korean national health insurance system and to present the actual costs of FELD to support a realistic reimbursement. In this regard, we included homogeneous data from a university hospital that strictly follows the regulations of the national health insurance system and minimizes the use of un-insured surgical materials. Nonetheless, a comparative study should have been attempted to truly determine the utility of the FELD procedure. We have acknowledged this limitation and described it in the limitations section.

Page 17, line 8

This study has several limitations. First, it presents results from a small number of patients, which were not sufficient to fully represent the cost-utility of FELD. In addition, this study analyzed only direct medical costs, although indirect medical costs may increase medical expenditures as a whole. However, the main purpose of this study was to show the relevance of the costs incurred at hospitals for FELD. In this regard, we used homogeneous data from patients who underwent the same surgical procedure and were managed with the same clinical pathway in a single tertiary referral hospital. Although the number of patients was small, the surgical technique, medical costs, and postoperative management were homogeneous. In addition, the surgery was performed at a university hospital, and no additional uninsured surgical materials such as a hemostatic agent or anti-adhesive agent were used, except for the electrode for FELD. We acknowledge that the small number of patients was the major weakness of this study, but the homogeneity may compensate for this limitation.

Second, spine surgery is performed at a wide range of hospitals, and the direct costs may be variable across hospitals and countries.[8] The current example of direct medical costs may not be representative, and it limits the generalizability of the findings. If the current suggestion for a practical reimbursement is accepted, data from multiple types of hospitals must be collected to set a realistic reimbursement amount. Last, but not least, FELD was not compared to standard open discectomy, which would have made it possible to truly determine the utility of FELD. However, open discectomies were performed at several hospitals, and the direct medical costs such as hospital stay, room charges, magnetic resonance imaging, and uninsured surgical materials were variable. Therefore, the need to preserve homogeneity meant that open discectomy could not be compared with FELD in this study.

Journal Requirements:

When submitting your revision, we need you to address these additional requirements.

1. Please ensure that your manuscript meets PLOS ONE's style requirements, including those for file naming. The PLOS ONE style templates can be found at 
<https://journals.plos.org/plosone/s/file?id=wjVg/PLOSOne_formatting_sample_main_body.pdf> and 
<https://journals.plos.org/plosone/s/file?id=ba62/PLOSOne_formatting_sample_title_authors_affiliations.pdf>

2. We note that the grant information you provided in the ‘Funding Information’ and ‘Financial Disclosure’ sections do not match.

When you resubmit, please ensure that you provide the correct grant numbers for the awards you received for your study in the ‘Funding Information’ section.

3. Thank you for stating the following in the Competing Interests section: "The first author (CHK) is a consultant of RIWOspine GmBH. All the authors declare that they have no conflicts of interest concerning the materials/methods used in this study or the findings described in this paper."

Please confirm that this does not alter your adherence to all PLOS ONE policies on sharing data and materials, by including the following statement: "This does not alter our adherence to  PLOS ONE policies on sharing data and materials.” (as detailed online in our guide for authors <http://journals.plos.org/plosone/s/competing-interests)>.  If there are restrictions on sharing of data and/or materials, please state these. Please note that we cannot proceed with consideration of your article until this information has been declared.

Please include your updated Competing Interests statement in your cover letter; we will change the online submission form on your behalf.

4. In your Data Availability statement, you have not specified where the minimal data set underlying the results described in your manuscript can be found. PLOS defines a study's minimal data set as the underlying data used to reach the conclusions drawn in the manuscript and any additional data required to replicate the reported study findings in their entirety. All PLOS journals require that the minimal data set be made fully available. For more information about our data policy, please see <http://journals.plos.org/plosone/s/data-availability>.

Upon re-submitting your revised manuscript, please upload your study’s minimal underlying data set as either Supporting Information files or to a stable, public repository and include the relevant URLs, DOIs, or accession numbers within your revised cover letter. For a list of acceptable repositories, please see <http://journals.plos.org/plosone/s/data-availability#loc-recommended-repositories>. Any potentially identifying patient information must be fully anonymized.

Important: If there are ethical or legal restrictions to sharing your data publicly, please explain these restrictions in detail. Please see our guidelines for more information on what we consider unacceptable restrictions to publicly sharing data: <http://journals.plos.org/plosone/s/data-availability#loc-unacceptable-data-access-restrictions>. Note that it is not acceptable for the authors to be the sole named individuals responsible for ensuring data access.

We will update your Data Availability statement to reflect the information you provide in your cover letter.

5. We note that you have stated that you will provide repository information for your data at acceptance. Should your manuscript be accepted for publication, we will hold it until you provide the relevant accession numbers or DOIs necessary to access your data. If you wish to make changes to your Data Availability statement, please describe these changes in your cover letter and we will update your Data Availability statement to reflect the information you provide.

6. Please include captions for your Supporting Information files at the end of your manuscript, and update any in-text citations to match accordingly. Please see our Supporting Information guidelines for more information: <http://journals.plos.org/plosone/s/supporting-information>.
