# Supplementary material for: Cost-utility analysis of endoscopic lumbar discectomy following a uniform clinical pathway in the Korean national health insurance system
Source: PLoS One. 2023 Jun 15;18(6):e0287092. doi: 10.1371/journal.pone.0287092 (PMC10270587; doi:10.1371/journal.pone.0287092)
Supplement: Attachment — Submitted filename: Revision letter 20230416.docx [file pone.0287092.s003.docx]

Author’s response to reviewers.

Reviewer #2: Thank you for adressing the issues and for a thorough revision. I support publication.

Congrats with your work!

Answer.

I appreciate the valuable comments and those were helpful in improving this paper. Thank you very much.

Reviewer #3: Dear Authors
I find that you have addressed a few of the queries raised in the previous round of review. Having looking into your intervention cohort, i find a significant number of patient also underwent open discectomy with follow-up data as given in Figure file. I urge the authors to include them into the analysis and make the study robust since the current analysis suffers from low number of patients included for analysis and lack of comparison group. Moreover, for inclusion into any health system a comparative analysis of the existing standard to the newer technique needs to be presented for acceptance.

Answer.

I highly appreciate your advice and the comparison between endoscopic surgery and the standard open discectomy will make this study robust. Although comparing cost-utility between full endoscopic lumbar discectomy (FELD) and open discectomy may have been the best study design, patients with open discectomy did not follow the clinical pathway and underwent surgeries in other hospitals. To maintain the homogeneity of data, we only analyzed patients with FELD, who underwent surgery in a non-profit tertiary hospital with a uniform clinical pathway.

During revision of manuscript, we tried to obtain cost data of patietns with open discectomy, but institutional review board did not approve to open sensitive cost data to the other hospital, because this study was a post hoc analysis and information of cost had not been planned from the beginning. Because special single-used surgical instruments are necessary for FELD, we should show the practical economical burden of FELD. Although this study included a small number of patients, the care of patients were standardized using a uniform clinical pathway and the direct cost was minimized in a non-profit tertiary hospital. Therefore, this study may be meaningful in showing the minimal burden of surgical cost and instruments of FELD. The current study may be referenced in countries with national health insruace to embrace economical burden of the hospital and patients by government. Nonetheless, we acknowledged the limitation and clarified it in the limitation section as followed.

Page 21, line 11

First, it presents results from a small number of patients without control group, which were not sufficient to fully represent the cost-utility of FELD. In addition, this study analyzed only direct medical costs, although indirect medical costs may increase medical expenditures as a whole. **However, the main purpose of this study was to show the relevance of the costs incurred at hospitals for FELD. In this regard, we used homogeneous data from patients who underwent the same surgical procedure and were managed with the same clinical pathway in a single non-profit tertiary referral hospital. Although the number of patients was small, the surgical technique, medical costs, and postoperative management were homogeneous. In addition, no additional uninsured surgical materials such as a hemostatic agent or anti-adhesive agent were used, except for the electrode for FELD. We acknowledge that the small number of patients was the major weakness of this study, but the homogeneity may compensate for this limitation.**

Reviewer #4: I congratulate the authors for the research work. The article tries to address one of the important bottlenecks in adapting - the cost of the procedure. However the article falls short of providing good scientifically relevant information for the readers. The important conclusion of the article is that the cost per QALY of FELD is less than the GDP per capita of ROK. This is more like a recommendation to a health administration rather than a scientific material for publication.

The authors have compared the outcomes of FELD with open discectomy right from the introduction. The article would have been more informative if the cost per QALY of both these were compared. I strongly recommend the authors to include the cost per QALY of the open discectomy as well.

Answer.

I highly appreciate your advice and the comparison between endoscopic surgery and the standard open discectomy will make this study robust. The purpose of this study was to show a cost-utility of full endoscopic lumbar discectomy (FELD) and showed an example of assessing cost-utility by using GDP of ROK. Nonetheless of the purpose, the current study may have limitation in not including control group (standard open discectomy). Although comparing cost-utility between FELD and open discectomy may have been the best study design, patients with open discectomy did not follow the clinical pathway and underwent surgeries in other hospitals. To maintain the homogeneity of data, we only analyzed patients with FELD, who underwent surgery in a non-profit tertiary hospital with a uniform clinical pathway.

During revision of manuscript, we tried to obtain cost data of patietns with open discectomy, but institutional review board did not approve to open sensitive cost data to the other hospital, because this study was a post hoc analysis and information of cost had not been planned from the beginning. Because special single-used surgical instruments are necessary for FELD, we should show the practical economical burden of FELD. Although this study included a small number of patients, the care of patients were standardized using a uniform clinical pathway and the direct cost was minimized in a non-profit tertiary hospital. Therefore, this study may be meaningful in showing the minimal burden of surgical cost and instruments of FELD. The current study may be referenced in countries with national health insruace to embrace economical burden of the hospital and patients by government. Nonetheless, we acknowledged the limitation and clarified it in the limitation section as followed.

Page 21, line 11

First, it presents results from a small number of patients without control group, which were not sufficient to fully represent the cost-utility of FELD. In addition, this study analyzed only direct medical costs, although indirect medical costs may increase medical expenditures as a whole. **However, the main purpose of this study was to show the relevance of the costs incurred at hospitals for FELD. In this regard, we used homogeneous data from patients who underwent the same surgical procedure and were managed with the same clinical pathway in a single non-profit tertiary referral hospital. Although the number of patients was small, the surgical technique, medical costs, and postoperative management were homogeneous. In addition, no additional uninsured surgical materials such as a hemostatic agent or anti-adhesive agent were used, except for the electrode for FELD. We acknowledge that the small number of patients was the major weakness of this study, but the homogeneity may compensate for this limitation.**

Methods: this study is a subgroup analysis of a previous study. Lines 1 to 9 of page 7 can be removed.
Line 16 page 9: Purpose of the study should be at the end of introduction and should be clear.

Answer.

I appreciate your comments. I deleted the paragraph and changed the purpose of this study in the introduction section as follows.

Page 6, line 6

The choice among surgical options would be fairer if the actual costs of those supplies were reimbursed, but WTP for FELD has not yet been determined in the ROK.[8-10] To address this gap, we provided a cost-utility of FELD and suggested an appropriate cost for reimbursement of the supplies used in FELD.[11]

Results are well written

Answer.

I appreciate your encouragement.

Discussion:
What is the additional relevant information provided in this study when compared to the Choi et al or Kim et al study? (reference 8 and 12). this should be included in discussion.

Answer.

I appreciate the critical comment. The data of Choi and Kim study were from both private hospital and university hospital, and the actual costs are different between private and university hospital. The purpose of this study was to suggest a practical reimbursement cost for FELD using a standardized clinical pathway while minimizing the use of uninsured surgical instruments. In this regards, we included patients in the non-profit tertiary referral hospital. Therefore, the homogeneity of data was the strength of this study. We clarified it in the limitation section as follows.

Page 22, line 11

**However, the main purpose of this study was to show the relevance of the costs incurred at hospitals for FELD. In this regard, we used homogeneous data from patients who underwent the same surgical procedure and were managed with the same clinical pathway in a single non-profit tertiary referral hospital. Although the number of patients was small, the surgical technique, medical costs, and postoperative management were homogeneous. In addition, no additional uninsured surgical materials such as a hemostatic agent or anti-adhesive agent were used, except for the electrode for FELD. We acknowledge that the small number of patients was the major weakness of this study, but the homogeneity may compensate for this limitation.**

Line 29, page - correct FEED to FELD
Answer

I am sorry for typo error. It was corrected.

General discussion on the method used for calculation of Cost per QALY should be added along with the comparison of other methods available.

Answer.

I am sorry for not explaing the method of cost-utility analysis. Usually, economical aspect of surgical technique is evaluated by caculating the cost to increase a unit of health outcome measurement. The outcome measurement includes quality of life, pain, disability, reoperation or adverse event. The current study used a quality adjusted life year (QALY) for health outcome measurement, which was the most common measurement for cost-utility analysis.

Page 19, line 3

**In evaluating a new surgical technique, the incurred cost to improve a unit in health outcome measurement is a common method in evaluating the technique under the health insurance system.[35,36] Utility measures varied between the EQ-5D and variations of the Short-Form Health Survey.[35] Effect measures varied widely between Visual Analogue Scale for pain, Neck Disability Index, Oswestry Disability Index, reoperation rates and adverse events.[35] The current study used EQ-5D for cost-utility analysis. The analysis showed the cost to improve a perfect 1-year quality of life per person (quality adjusted life year, QALY), which was the most common measurement for cost-utility analysis.**

Limitations:
lines 17,18 page 17 are getting repeated and can be removed.

Answer.

The repeated sentence was removed.

lines 26,27 page 17, in the parent study of this study, there are patients who underwent open discectomy. Weren't operated in the same hospital? Comparing them for cost utility will give homogeneity.

Answer.

I appreciate your comment. FELD was performed in our hospital and open discectomy was performed in the other hospital. Therefore, patients with open discectomy did not follow the clinical pathway of FELD. In addition, data of medical cost was not allowed to review without pre-planned study in the other hospital. As we noted, the main purpose of this study was to suggest a practical imbursement cost for FELD. We hope the current method would be appropriate for the purpose of this study.
