# Supplementary material for: Cost-utility analysis of endoscopic lumbar discectomy following a uniform clinical pathway in the Korean national health insurance system
Source: PLoS One. 2023 Jun 15;18(6):e0287092. doi: 10.1371/journal.pone.0287092 (PMC10270587; doi:10.1371/journal.pone.0287092)
Supplement: Attachment — Submitted filename: Revision letter 2020525.docx [file pone.0287092.s004.docx]

Author’s response to reviewers.

Additional Editor Comments:

Dear Authors
I see that you have acknowledged the limitation pointed out in the discussion of the manuscript. You could also stress upon the need for a common surgical pathway for all the cases to make future assessments of evolving surgical techniques and methods feasible. Hence, I suggest acceptance of the present version following incorporation of the suggested minor edits to the limitation to stress the need for common surgical pathway.

Answer.

Thank you for the positive comments for the revised manuscript. A standardization of surgical pathway will improve cost-utility of surgical treatment. This study showed the example and I appreciate the comment. We emphasized the importance of common surgical pathway like followings in the limitation section.

Page 23, line 8

**A common surgical pathway in care of patients and using surgical materials are required to incorporate FELD as one of appropriate surgical options.**

Reviewer #2: All comments were addressed so the paper can be accepted
All comments were addressed so the paper can be accepted

Answer.

Thank you for the positive comments for the revised manuscript. A standardization of surgical pathway will improve cost-utility of surgical treatment. This study showed the example and I appreciate the comment. We emphasized the importance of common surgical pathway like followings in the limitation section.

Page 23, line 8

**A common surgical pathway in care of patients and using surgical materials are required to incorporate FELD as one of appropriate surgical options.**

Reviewer #3: Dear Authors
I see that you have acknowledged the limitation pointed out in the discussion of the manuscript. You could also stress upon the need for a common surgical pathway for all the cases to make future assessments of evolving surgical techniques and methods feasible. Hence, I suggest acceptance of the present version following incorporation of the suggested minor edits to the limitation to stress the need for common surgical pathway.

Answer.

Thank you for the positive comments for the revised manuscript. A standardization of surgical pathway will improve cost-utility of surgical treatment. This study showed the example and I appreciate the comment. We emphasized the importance of common surgical pathway like followings in the limitation section.

Page 23, line 8

**A common surgical pathway in care of patients and using surgical materials are required to incorporate FELD as one of appropriate surgical options.**
